# A Case of Acute Myeloid Leukemia Mimicking Blastic Plasmacytoid Dendritic Cell Neoplasm: Utility of the Proposed Upcoming WHO-5 Diagnostic Criteria

**DOI:** 10.1155/2023/5014728

**Published:** 2023-11-01

**Authors:** Bhvaneet Jhajj, Ryan Henrie, Youness El-Khalidy, Habib Moshref Razavi

**Affiliations:** ^1^Division of Hematopathology, Royal Columbian Hospital, New Westminster, BC, Canada; ^2^Department of Pathology and Laboratory Medicine, University of British Columbia, Vancouver, BC, Canada; ^3^Division of Hematology, Department of Medicine, University of British Columbia, Vancouver, BC, Canada

## Abstract

Blastic plasmacytoid dendritic cell neoplasm (BPDCN) is an aggressive hematologic malignancy which is associated with a distinctive morphologic appearance. However, the morphology is not specific, and diagnostic characterization requires integration of immunophenotypic and genetic testing. We herein report a case of a 35-year-old female patient who presented with worsening cytopenia. A bone marrow aspirate identified medium-sized blastic cells with perinuclear microvacuoles (“pearl neckless”). Occasional blasts demonstrated a “hand mirror” appearance. Tandem flow cytometry showed an atypical population of dim CD45 events with expression of CD4, CD56, CD117, CD123, and monocytic markers such as CD64. Fluorescence in situ hybridization (FISH) showed evidence of a KMT2A rearrangement with an unknown partner on chromosome 19. Expression of MPO and muramidase was present. The final diagnosis was acute monocytic leukemia (AMoL). Due to the overlapping features of acute myeloid leukemia and BPDCN, the 5^th^ Edition of the World Health Organization (WHO) Classification of Haematolymphoid Tumours provides new criteria for the diagnosis of BPDCN. Our case highlights the utility of these criteria.

## 1. Introduction

BPDCN and AMoL are both aggressive hematologic malignancies with distinct prognoses and treatments [1]. BPDCN is a historically rare diagnosis that often presents with skin lesions [1]. The differential diagnosis includes acute myeloid leukemia, particularly AMoL [2]. Distinguishing these entities can represent a diagnostic challenge. We describe a case of AMoL where the initial pathologic appearance raised suspicion of BPDCN and highlight the diagnostic work-up that facilitated accurate diagnostic classification.

## 2. Case Presentation

**Figure 1:**
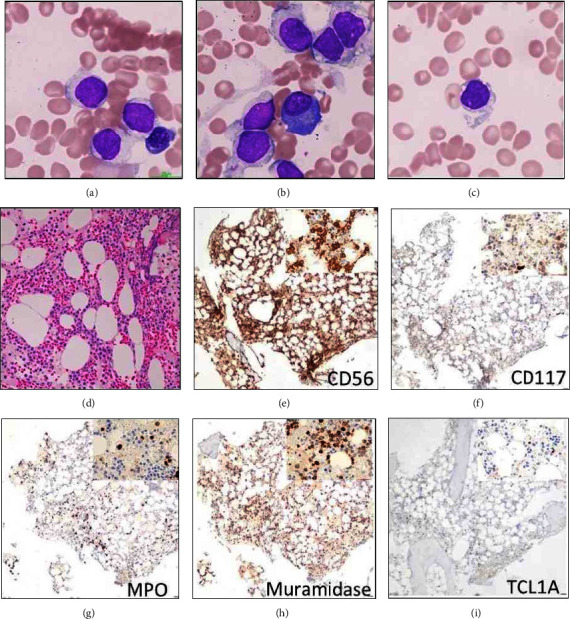
Morphologic findings of the bone marrow aspirate. The infiltrate of medium-sized blastic cells with perinuclear microvacuoles (“pearl neckless”), eccentric nuclei, fine chromatin texture, and few nucleoli is demonstrated on the upper three panels (Wright Giemsa stain, ×50 magnification). H&E and immunohistochemical staining findings are demonstrated in the lower 6 panels (×20 magnification).

A 35-year-old female patient presented to our hospital with pancytopenia (hemoglobin 88 g/L, platelets 64 × 10^9^/L, and neutrophils 0.2 × 10^9^/L). As her symptoms included weight loss and intermittent night sweats, a bone marrow biopsy was performed to rule out a pathological basis for her presentation. The patient did not demonstrate any notable dermatologic lesion. The bone marrow aspirate showed an infiltrate of medium-sized blastic cells with perinuclear microvacuoles (“pearl neckless”), eccentric nuclei, fine chromatin texture, and few nucleoli. These blastic cells occasionally resembled “hand mirrors” (Figures 1(a)–1(c); upper panels). These morphologic findings were suspicious for BPDCN. A hypocellular biopsy revealed a monomorphic cellular infiltrate with abundant eosinophilic cytoplasms (Figures 1(d)–1(i); lower panels).

**Figure 2:**
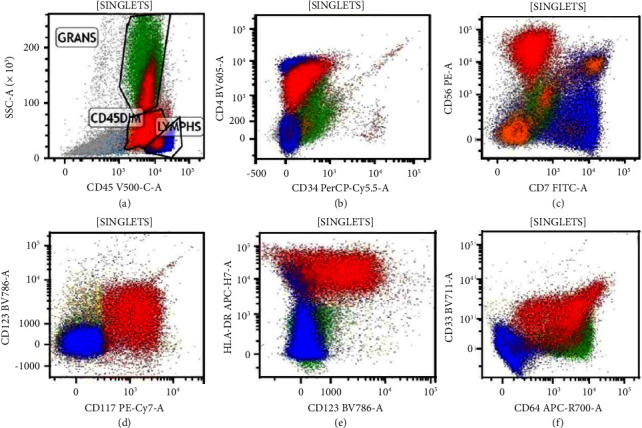
Flow cytometry of the bone marrow aspirate. The neoplastic population is coloured in red and demonstrates positivity for dim CD45, CD4, CD56, CD117, CD123, and CD64.

By immunohistochemistry, the malignant cells expressed CD56, muramidase (lysozyme), partial/dim myeloperoxidase, and CD117. TCL1A stain was negative. Tandem flow cytometry showed an atypical population of dim CD45 events with expression of CD4, CD56, CD117, CD123, and CD64 (Figures 2(a)–2(f); Crimson population).

**Figure 3:**
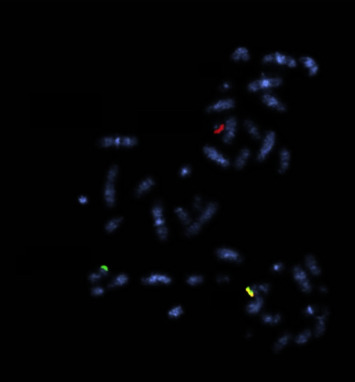
Fluorescence in situ hybridization (FISH) showing the evidence of a KMT2A rearrangement (break-apart probes; normal KMT2A indicated by a yellow fusion signal. Red and green signals imply translocation/break-apart).

**Figure 4:**
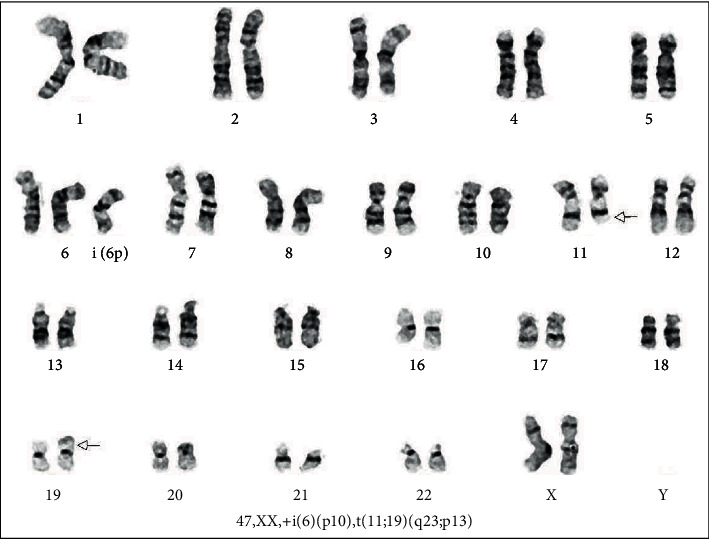
Karyotype demonstrating a *t*(11; 19) translocation (derivative chromosomes indicated with arrows) as well as an isochromosome 6p.

Fluorescence in situ hybridization (FISH) showed the evidence of a KMT2A rearrangement on chromosome 11 (q23) confirming the cytogenetic impression of *t*(11; 19). The current study did not identify the exact partner gene (candidates include ELL at 19p13.1 or MLLT1 at 19p13.3; Figures 3 and 4, respectively).

Given the lack of cutaneous findings, presence of muramidase and MPO expression (not expected in BPDCN), and absence of TCL1A expression (expected in BPDCN), the final diagnostic classification was AMoL.

This patient was treated with induction chemotherapy including seven days of cytarabine and three days of daunorubicin. She proceeded to consolidation with high-dose cytarabine, complicated by febrile neutropenia which required antibiotic therapy. She subsequently proceeded to an allogeneic stem cell transplant from a fully HLA-matched donor with myeloablative conditioning with busulfan and cyclophosphamide. Antithymocyte globulin was given as prophylaxis for graft versus host disease.

On day 90 following her transplant, she developed hypoxemic respiratory failure with bilateral pulmonary infiltrates. Respiratory samples showed positivity for rhinoviruses and enteroviruses. Her respiratory status deteriorated over the following days despite aggressive supportive care in the intensive care unit. She suffered a cardiac arrest from marked hypoxemic and could not be resuscitated.

## 3. Discussion

The presented case demonstrates the overlapping histologic features of BPDCN and AMoL. Blasts showing a “pearl necklace” appearance, eccentric nuclei, fine chromatin texture, and few nucleoli initially generated suspicion for BPDCN. However, several clinical, genetic, and immunophenotypic features allowed appropriate classification as AMoL.

Clinically, cutaneous involvement appears to be more common in BPDCN than AMoL [2] and was not noted in our case. There are overlapping immunohistological features such as expression of CD4, CD56, and CD123 [2]. Expressions of all of these markers were noted in this case. However, the monocytic marker CD64 is found as well, which favors AMoL rather than BPDCN [2]. MPO expression, as found in the patient, is found more commonly in AMoL than in BPDCN [1]. In terms of molecular testing, the KMT2A rearrangement on chromosome 11, as demonstrated by FISH, is another commonality between the conditions [4, 5]. However, this tends to be more characteristic of AMoL, while the more frequent genetic alterations in BPDCN include deletions or mutation of the genes CDKN2A/CDKN2B, RB1, CDKN1B, LATS2, and IKZF1 [6].

The forthcoming 5^th^ edition of the WHO Classification of Haematolymphoid Tumours provides new immunophenotypic diagnostic criteria for the diagnosis of BPDCN [7]. These delineate expected positive and negative markers in BPDCN. The suggested diagnosis criteria based on immunophenotyping requires expression of CD123 and one other pDC marker (e.g., TCF4, TCL1, CD303, or CD304) in addition to CD4 and/or CD56, or, expression of any three pDC markers and absent expression of all expected negative markers (e.g., CD3, CD14, CD34, lysozyme, and MPO). Although some expected positive markers were expressed (CD123, CD4, CD56, by flow cytometry), multiple expected negative markers were also present (MPO, lysozyme) capturing the diagnosis for AMoL by the new/suggested criteria [7].

In summary, AMoL and BPDCN have overlapping histologic features, making it challenging to distinguish them pathologically. This case highlights the overlap between these entities and the utility of the updates for the diagnosis of BPDCN in the new WHO Classification of Haematolymphoid Tumours. The case also demonstrates the importance of integrating clinical information, immunophenotype, and genetic studies for accurate classification.

## Data Availability

All data used in this article are available for review.
